# Unraveling migratory corridors of loggerhead and green turtles from the Yucatán Peninsula and its overlap with bycatch zones of the Northwest Atlantic

**DOI:** 10.1371/journal.pone.0313685

**Published:** 2024-12-06

**Authors:** Elizabeth Labastida-Estrada, Salima Machkour-M’Rabet

**Affiliations:** 1 Departamento de Zoología, Instituto de Biología, Universidad Nacional Autónoma de México, Mexico City, Mexico; 2 Laboratorio de Ecología Molecular y Conservación, El Colegio de la Frontera Sur, Chetumal, Quintana Roo, Mexico; MARE – Marine and Environmental Sciences Centre, PORTUGAL

## Abstract

Bycatch represents a conservation problem when endangered species are affected. Sea turtles are highly vulnerable to this threat as their critical habitats overlap with fishing zones in all regions of the world. We used sequences of the mitochondrial DNA control region obtained from loggerhead (*Caretta caretta*) and green (*Chelonia mydas*) turtles to determine the migratory routes between nesting habitats in the Yucatán Peninsula and their critical marine habitats in the Northwest Atlantic. Mixed Stock Analysis revealed that loggerheads from Quintana Roo migrated to foraging areas in the northwestern Atlantic. Migratory routes used by green turtles are determined by their natal nesting colony: (1) green turtles from the Gulf of Mexico migrate to foraging aggregations in Texas and the northern Gulf of Mexico, (2) Mexican Caribbean turtles travel to foraging grounds in Florida, and (3) a smaller proportion of individuals born in the Yucatán Peninsula display a local connectivity pattern. Our results suggest that the migratory corridors used by Mexican loggerheads overlap with longline fisheries in the mid-Atlantic where sea turtle bycatch is comprised predominantly of immature individuals. Green turtles from the Yucatán Peninsula migrate to critical habitats that overlap with shrimp trawl fisheries within the Gulf of Mexico. Bycatch data and the identification of migratory corridors used by loggerheads and green turtles suggests that shrimp trawl fisheries on the east coast of the U.S. and the Gulf of Mexico pose a serious threat to the conservation and recovery of Mexican sea turtle populations.

## Introduction

Historically, sea turtles were extensively harvested by fisheries worldwide [1, 2]. Large-scale commercial fishing of sea turtles reported a global capture of over 17,000 tons during the late 1960s [3]. In Mexico, sea turtle commercial fisheries increased by 633% between 1962 and 1967, and it was estimated that a total of 14,500 tons were captured in 1968 [1, 4]. Overexploitation contributed to the significant decline of sea turtle populations worldwide [3] and led to the protection of sea turtles through legal restrictions that banned their commercial catch in most countries [5]. International instruments such as the Red List of Threatened Species from the International Union for Conservation of Nature (IUCN) and the Convention of International Trade in Endangered Species of Wild Fauna and Flora (CITES) have played a critical role in the legal protection of all species of sea turtles and their populations [5, 6]. Furthermore, various countries have implemented their own legal mechanisms for the protection of sea turtles. For example, the Mexican government decreed a permanent fishery ban on sea turtles in 1990, and the NOM-059-SEMARNAT-2010 listed all six species of sea turtles under the ’endangered’ category, which is the category of highest risk among the four existing ones (probably extinct in the wild, endangered, threatened, and subject to special protection) [7].

Legal restrictions and several decades of conservation efforts have resulted in the recovery of many sea turtle populations worldwide [8, 9]. However, bycatch, here defined as the unintentional capture of these species during fishing operations, continues to pose a significant threat to their populations [10]. Marine megafauna, such as sea turtles, are especially vulnerable to bycatch because they inhabit habitats that span extensive areas across different geographical and political borders, as well as unique oceanographic regions [10]. The impact of bycatch on sea turtle populations depends on several life-history characteristics (e.g., slow growth rates and late sexual maturity) [11], as well as on the spatial-temporal overlap of fisheries with critical habitats used by different populations [12, 13].

Commonly, the effect of bycatch has been assessed at a global scale, which makes it difficult to propose strategies at a regional or local scale [12]. In this context, Wallace et al. [14, 15] proposed that the evaluation of threats, including bycatch, should be conducted within conservation units called Regional Management Units (RMUs). The RMUs for sea turtles represent conservation units that differ in genetics, distribution, movements, and demography [14, 15]. Each RMU includes multiple Management Units (MUs) defined as nesting colonies that are demographically independent and exhibit significant differences in their genetic frequencies [16, 17]. Komoroske et al. [16] suggested that the MUs represent the appropriate scale for monitoring population dynamics, and particularly their vulnerability to threats.

The loggerhead turtle (*Caretta caretta* Linnaeus, 1758) is globally distributed across subtropical and temperate regions, comprising 10 RMUs over a wide geographic range [15], and is listed as Vulnerable on the IUCN Red List [18]. On the other hand, the green turtle (*Chelonia mydas* Linnaeus, 1758) has a circumglobal distribution, with its global population divided into 11 distinct RMUs [15], and is classified as Endangered on the IUCN Red List [19]. The Northwest Atlantic (NWA) constitutes an important RMU that hosts nesting populations and critical marine habitats of loggerhead and green turtles throughout the Atlantic coast, Gulf of Mexico, and the Caribbean Sea [14, 15]. For loggerhead turtles, the NWA RMU consists of representative MUs located on the Atlantic coasts of the U.S. in Florida, Georgia, and North Carolina, as well as on the Quintana Roo coast in Mexico according to the Turtle Expert Working Group TEWG [20]. Concerning green turtles, the NWA RMU encompasses nesting colonies from Tortuguero in Costa Rica, the Yucatán Peninsula in Mexico, and the East coast of Florida in the U.S. [21]. The waters from NWA are suitable habitats for development, foraging, and migratory movements for both species [22]. Coastal zones in Florida, Texas, and the Yucatán Peninsula host mixed aggregations consisting of individuals from different nesting populations and therefore play a critical role in the recovery and conservation of these populations [21].

However, these critical habitats used by loggerhead and green turtles within the NWA overlap with commercial fisheries such as shrimp trawls, shark bottom longline, and pelagic longline that target migratory species such as tuna and swordfish [23, 24]. According to bycatch estimations based on onboard observer data obtained from 22 shrimp trawl fisheries in the Gulf of Mexico and southern Atlantic, 1,827 sea turtles were caught between 2014 and 2015, including 388 green and 315 loggerheads turtles [24]. Pelagic longline fisheries from the Atlantic and Gulf of Mexico reported a bycatch of 2,176 sea turtles, of which 501 were loggerheads, during the same period [24].

To assess the effect of bycatch on sea turtle populations, we need to understand how their critical habitats are interconnected and identify migratory corridors where the risk of bycatch mortality or other detrimental interactions could potentially be higher [25–27]. Movement patterns and migratory corridors between critical habitats have been identified using several approaches, including tag returns, satellite tracking, stable isotope analysis, molecular markers, and dispersal simulations [28–31]. Even though satellite telemetry is very efficient, its high operational cost frequently leads to analyses based on small sample sizes [28]. Alternatively, the molecular approach provides complementary information to other tracking techniques and can offer robust insights into the migratory routes and movement patterns of sea turtles [32].

Mitochondrial DNA (mtDNA) haplotypes constitute a unique signature that is inherited maternally, enabling the identification of nesting populations and their geographical distribution, as well as the likely origins of adults and juveniles in marine habitats [33]. Mixed Stock Analysis (MSA) is a Bayesian method that uses mtDNA haplotypes to simultaneously estimate the origin and destination of individuals in a metapopulation shaped by multiple source populations and multiple mixed stocks (foraging aggregations, breeding areas, or migratory corridors) [34]. This approach has been applied to predict sea turtle movements between their critical habitats, providing insights into the spatial ecology and connectivity of these migratory species [16, 34]. It has also been used to determine the source nesting populations of sea turtles caught in fisheries areas in the NWA [25–27, 35].

The Yucatán Peninsula and its offshore regions host important nesting colonies of loggerhead and green turtles, as well as critical habitats for their development, foraging, and migratory corridors [31, 36]. Determining the contribution of loggerhead and green turtles from the Yucatán Peninsula to neritic and oceanic habitats within the NWA through Mixed Stock Analysis will allow us to infer the migratory corridors used by these species. Additionally, this study provides new data about bycatch as a potential threat to sea turtles within these habitats, which could help improve and enhance recovery and conservation strategies. Therefore, the aims of this study are: 1) to define the MUs of loggerhead and green turtles for nesting colonies in the Yucatán Peninsula, 2) to infer potential migratory corridors between the nesting habitats of these species and their critical marine habitats in the NWA, and 3) to identify the potential spatial overlap between migration corridors used by loggerhead and green turtles from the Yucatán Peninsula and the fishing zones within the NWA.

## Materials and methods

### Ethical statements

The activities of capture, sampling, and monitoring were performed by qualified staff and were authorized by the legal permits SGPA/DGVS/08337/15 and SGPA/DGVS/06013/16 All biological samples used in this study were collected under the permits mentioned above. In addition, this research was approved by the “Comité de Ética para la Investigación (CEI)” of El Colegio de la Frontera Sur.

### Sample collection

**Fig 1:**
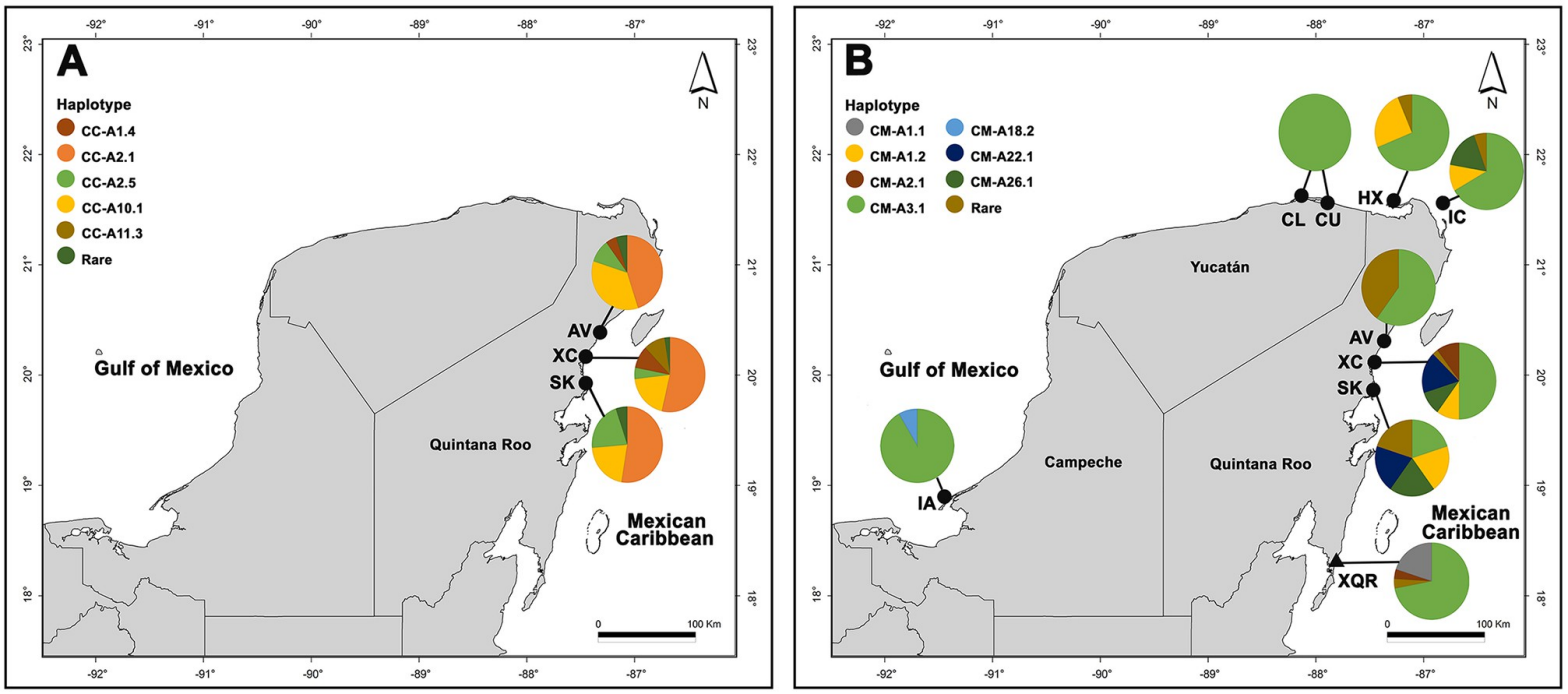
Localities sampled in the Yucatán Peninsula (Mexico) and mtDNA control region haplotype frequency for each (pie chart). (A) Loggerhead turtle nesting colonies (circles) from Quintana Roo. (B) Green turtle nesting colonies (circles) and a foraging aggregation (triangle) from the Yucatán Peninsula. See Table 1 for the abbreviation of each locality.

During the nesting seasons (from May to October) of 2015 and 2016, 93 tissue samples were collected from female loggerhead turtles from three nesting colonies (Table 1 and Fig 1A) and 165 tissue samples were collected from female green turtles from eight nesting colonies along the Yucatán Peninsula coast (Table 1 and Fig 1B). Furthermore, we included 26 tissue samples of immature green turtles from a foraging aggregation within the Parque Nacional Arrecifes de Xcalak in Quintana Roo, collected by researchers from the “Instituto Tecnólogico de Chetumal” (Table 1 and Fig 1B). It is worth noting that prior to this study, no foraging aggregations of green turtles from the Yucatán Peninsula had been genetically characterized.

**Table 1:** Sampled localities for loggerhead and green turtle nesting colonies and a green turtle foraging aggregation in the Yucatán Peninsula, Mexico.

Abbrev	Locality	N	Nesting season	Geographic coordinates
**Loggerhead turtle nesting colonies**				
AV	Aventuras DIF, Quintana Roo	23	2016	20°21’58” N, 87°19’54” W
XC	Xcacel-Xcacelito, Quintana Roo	50	2015, 2016	20°20’18” N, 87°20’53” W
SK^1^	Sian Ka’an, Quintana Roo	20	2016	21°30’36” N, 86°55’36” W
**Green turtle nesting colonies**				
IA	Isla Aguada, Campeche	24	2015, 2016	18°47’31” N, 91°29’46” W
CU	El Cuyo, Yucatán	26	2016	21°31’00” N, 87°40’10” W
CL	Las Coloradas, Yucatán	13	2016	21°33’17” N, 87°49’26” W
HX	Holbox, Quintana Roo	18	2016	21°33’33” N, 87°19’36” W
IC	Isla Contoy, Quintana Roo	21	2015	21°27’46” N, 86°47’07” W
AV	Aventuras DIF, Quintana Roo	5	2016	20°21’58” N, 87°19’54” W
XC	Xcacel-Xcacelito, Quintana Roo	51	2015, 2016	20°20’18” N, 87°20’53” W
SK^2^	Sian Ka’an, Quintana Roo	7	2016	20°05’09” N, 87°28’35” W
**Green turtle foraging aggregation**				
XQR	Xcalak, Quintana Roo	26	2016	18°17’27” N, 87°49’14” W

N: Sample size.

^1^ Includes the beaches of Cahpechen, Kanzul and Lirios Balandrin, Quintana Roo, Mexico.

^2^ Includes the beaches of Kanzul and Cahpechen, Quintana Roo.

Tissue samples were obtained from the edge of the front flipper using sterile 3 mm biopsy punches and preserved in a salt-saturated 20% DMSO solution at 4°C until molecular analysis. All individuals were marked with Inconel tags (National Band and Tag Co. 681) on the right flipper for identification and to avoid duplicate samples.

### DNA extraction and PCR amplification

Genomic DNA was obtained using the Wizard^®^ Genomic DNA Purification Kit (Promega) following the animal tissue protocol. Polymerase chain reaction (PCR) amplified a ~800 bp fragment of the mtDNA control region using the primers LCM15382 and H950g for both species [37]. The PCR amplification protocol was carried out according to Shamblin et al. [38]. The length of the amplified fragment was verified by electrophoresis on a 2% agarose gel in 1X TAE buffer using GelRed (Biotum) as staining method and a 100 bp DNA ladder (Promega) as a molecular weight marker. PCR products were purified and sequenced on an ABI 3730xl DNA analyzer.

### Genetic analysis

#### Haplotype assignment and identification of MUs of loggerhead and green turtles from the Yucatán Peninsula

Sequences were edited and aligned using Bioedit 7.2.5 [39]. Haplotypes were assigned based on reference sequences for the longer fragment of the mtDNA control region for loggerhead (~800 bp) and green turtles (~817 bp). These reference sequences are available at https://accstr.ufl.edu/resources/mtdna-sequences.

To determine the number of MUs of loggerhead and green turtle nesting colonies from the Yucatán Peninsula, the genetic differentiation between nesting colonies was evaluated via an Analysis of Molecular Variance (AMOVA; 10,000 permutations, *p*-value ≤ 0.05) in ARLEQUIN 3.5 [40] considering different scenarios: two scenarios for loggerhead nesting colonies in the Mexican Caribbean and four scenarios for green turtle nesting colonies in the Yucatán Peninsula (details of dataset and scenarios tested in S1 File, S1 and S2 Figs). Additionally, a pairwise *F*_ST_ test using haplotype frequencies was performed in ARLEQUIN 3.5 to support the genetic differentiation between grouping suggested via AMOVA.

#### Mixed Stock Analysis

The ’many-to-many’ Mixed Stock Analysis (MSA) was performed using the Mixstock package [34] in language R, allowing us to infer the migratory movements of loggerhead and green turtles born in the Yucatán Peninsula towards marine habitats within the NWA. The Gelman-Rubin diagnostic test was used to confirm the convergence of the chains; values ≤1.2 (after 100,000 MCMC) indicated the validity of the model [41]. For loggerhead turtles, we used haplotype frequencies from individuals caught incidentally in nine fishery areas of the NWA as ‘mixed stocks’ [25, 27, 42] (S1 Table and S3 Fig), and nine MUs identified via AMOVA as ‘source populations’ [43, 44] (S2 Table). As for green turtles, we included haplotype frequencies from seven foraging aggregations from the Mexican Caribbean, Florida, Texas, and northwestern Gulf of Mexico as ‘mixed stocks’ [32, 45–48] (S3 Table), and seven MUs identified via AMOVA as ‘source populations’ [38, 49] (S4 Table). Orphan haplotypes (haplotypes not identified in nesting colonies) were excluded and the estimated nesting abundance for each MU (estimated female number/year for each MU; S2 and S4 Tables) was included as an ecological covariate under the assumption that contributions are proportional to rookery size [34].

### Potential overlaps between fishery areas and sea turtle critical habitats in the NWA

We inferred the potential migratory corridors used by loggerhead and green turtles from the Yucatán Peninsula based on the estimated contributions of each MU to fishing areas for loggerhead turtles and to the foraging aggregations of green turtles in the NWA. The probable corridors between MUs and marine habitats were drawn based on ocean currents patterns recorded in the NWA during the sampling years (2015 and 2016), which were obtained from NASA’s OCEAN MOTION website via the Ocean Surface Current (OSCAR) data (https://www.oceanmotion.org/html/resources/oscar.htm).

To identify the zones where there is overlap between fishing areas and critical habitats used by loggerheads and green turtles in the NWA, we utilized available records of interactions between these species and the shrimp trawl fisheries in this region. These records were obtained by direct observations and compiled by the National Observer Program of the National Oceanic and Atmospheric Administration (NOAA) (https://apps-sefsc.fisheries.noaa.gov/apex/f?p=112:20). This analysis is focused exclusively on shrimp trawl fisheries for which data is available. Based on those data, we created maps in ArcGIS 10.2.1 that illustrate the geographical distribution of interactions between both species of sea turtle and shrimp trawl fisheries, as well as their overlap with migratory corridors previously inferred in the NWA region. All vector layers in the shapefile format used for the map backgrounds were downloaded for free from http://tapiquen-sig.jimdofree.com.

## Results

### Loggerhead turtles

Thirteen mtDNA haplotypes were identified from 91 of 93 sequences obtained from female loggerhead turtles from Quintana Roo nesting colonies. The most frequent haplotype was CC-A2.1 (45%) followed by CC-A10.1 (21%). Three haplotypes were less common: CC-A2.5 (9%), CC-A1.4 (5.5%), and CC-A11.3 (4.5%). The eight remaining haplotypes were less frequent (≤3%) and were considered rare (Table 2 and Fig 1A). For nesting colonies along the Quintana Roo coast, AMOVA results showed that both tested scenarios did not detect genetic differentiation between localities (S5 Table). Although the largest percentage of genetic variation was within populations (100%), this result was not significant (p = 0.53). In addition, the pairwise *F*_ST_ comparison results showed very low and non-significant values (S6 Table), suggesting that all nesting colonies from Quintana Roo be considered as a single MU, which will be referred to as QRMX.

**Table 2:** Mitochondrial DNA control region haplotype frequencies (~800 bp fragment) for the loggerhead turtle nesting colonies from the Yucatán Peninsula, Mexico.

Haplotype	AV	XC	SK	Total	Hap freq
CC-A1.3	-	1	-	1	1.1
CC-A1.4	1	4	-	5	5.5
CC-A2.1	9	22	10	41	45.0
CC-A2.3	1	-	1	2	2.2
CC-A2.5	2	2	4	8	9.0
CC-A3.1	-	2	-	2	2.2
CC-A5.1	-	1	-	1	1.1
CC-A8.1	1	1	1	3	3.0
CC-A9.1	1	2	-	3	3.0
CC-A10.1	7	8	4	19	21.0
CC-A11.3	-	4	-	4	4.5
CC-A.11.5	-	1	-	1	1.1
CC-A14.1	-	1	-	1	1.1
N	22	49	20	91	100%
N hap	7	12	5	13	

AV: Aventuras DIF, XC: Xcacel-Xcacelito, SK: Sian Ka’an, N: Sample size, N hap: number of haplotypes, Hap freq: Haplotype frequency (%).

Furthermore, our mtDNA sequences revealed two hybrid females, representing 2.2% of all sampled individuals. Both females displayed phenotypic characteristics indicative of loggerhead turtles (e.g., large head, with heavy strong jaws and reddish-brown carapace). However, genetically the first female from Xcacel-Xcacelito was a hybrid between a loggerhead and a hawksbill turtle (*Eretmochelys imbricata* Linnaeus, 1766) with haplotype EiA01. The second female from Aventuras DIF was identified as a hybrid between a loggerhead and a green turtle with haplotype CM-A16.1. The aforementioned genetic sequences were omitted from subsequent analyses.

**Fig 2:**
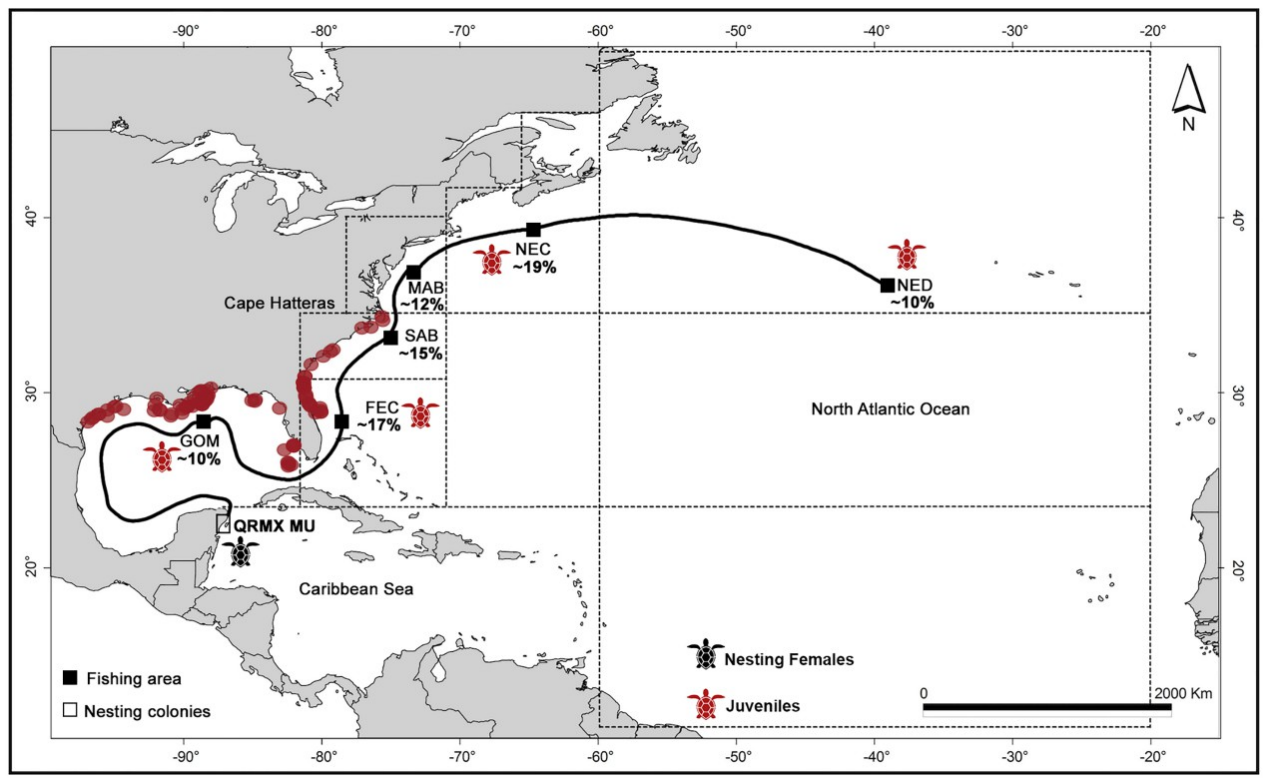
Migratory corridors in the NWA (black line) used by loggerhead turtles from Quintana Roo nesting colonies identified by MSA. Quintana Roo MU (QRMX MU) is delimited by a rectangle and the fishing areas are indicated by black squares: Gulf of Mexico (GOM), Florida east coast (FEC), South Atlantic bight (SAB), Mid Atlantic bight (MAB), Northeast coastal (NEC), and Northeast Distant Atlantic (NED). The contribution of loggerheads from QRMX MU to each fishing area is indicated in percentage. The brown dots indicate the geographic distribution of interactions between loggerhead turtles and shrimp trawl fisheries in the NWA (available data at https://apps-sefsc.fisheries.noaa.gov/apex/f?p=112:20) to identify the overlap between the fishing areas and migratory corridors of loggerhead turtles in the NWA. The life stage of loggerhead turtles in nesting colonies and fishing areas is indicated by turtle color. For data on the size of turtles, see S9 Table.

The MSA based on source nesting colonies from the NWA grouped into eight MUs (S2 Table) satisfied the Gelman–Rubin criterion, reporting a value of 1.02. MSA results showed that loggerhead turtles caught in six fishing areas from the NWA originated from the QRMX MU. The fishing areas with the highest percentage of Mexican loggerhead turtles were the NEC (19%), FEC (17%), and SAB (15%). The MSA results estimated a lower percentage of individuals caught in the MAB, NED, and GOM (ranging from 10 to 12% each). In the remaining areas, the bycatch of loggerhead turtles from QRMX MU was less than 6% (Fig 2).

Based on the data from direct observations compiled by the National Observer Program of the NOAA, we found 91 records of interactions between loggerhead turtles and shrimp trawl fisheries within the NWA, between February 2009 and June 2021. Most of these records (represented by brown dots in Fig 2) overlapped with the potential migratory routes identified in this study. These routes extend from the Texas to North Carolina coasts, and include the GOM, FEC, and SAB fishing areas.

### Green turtles

A total of 165 sequences were obtained from samples collected in eight nesting colonies from the Yucatán Peninsula, which were classified into 15 mtDNA haplotypes (Table 3 and Fig 1B). Haplotype CM-A3.1 was reported in high frequencies (66.7%) in all sampled localities while all others are present in low proportions (ranging from 6.7% to 0.6%). Six endemic haplotypes were identified in the Yucatán Peninsula: CM-A16.1, CM-A17.1, CM-A18.1, CM-A18.2, CM-A22.1, and CM-A26.1. El Cuyo and Las Coloradas reported a single haplotype (CM-A3.1) while Xcacel-Xcacelito reported eight, this being the largest number of haplotypes of all nesting colonies (Table 3 and Fig 1B). For the Xcalak foraging aggregation, we obtained 26 sequences that were classified into five mtDNA haplotypes (Table 4 and Fig 1B). Similar to the nesting colonies, the CM-A3.1 haplotype was the most frequent, representing 69.2% of the sample, with CM-A1.1 following at 19.2%, and others at very low frequencies. In this foraging aggregation, no orphan haplotypes were reported.

**Table 3:** Mitochondrial DNA control region haplotype frequencies (~817 bp fragment) for green turtle nesting colonies from the Yucatán Peninsula, Mexico.

Haplotype	IA	CU	CL	HX	IC	AV	XC	SK	Total	Hap freq
CM-A1.1	-	-	-	-	-	1	2	1	4	2.4
CM-A1.2	-	-	-	4	2	-	4	1	11	6.7
CM-A1.4	-	-	-	1	1	-	3	-	5	3.0
CM-A2.1	-	-	-	-	-	-	4	-	4	2.4
CM-A3.1	22	26	13	11	14	3	20	1	110	66.7
CM-A5.1	-	-	-	-	1	-	-	-	1	0.6
CM-A15.1	-	-	-	-	-	1	1	-	2	1.2
CM-A16.1	-	-	-	1	-	-	-	2	3	1.8
CM-A17.1	-	-	-	-	-	-	3	-	3	1.8
CM-A18.1	2	-	-	-	-	-	-	-	2	1.2
CM-A18.2	-	-	-	1	-	-	1	-	2	1.2
CM-A22.1	-	-	-	-	-	-	7	1	8	4.8
CM-A26.1	-	-	-	-	3	-	4	1	8	4.8
CM-A27.1	-	-	-	-	-	-	1	-	1	0.6
CM-A29.1	-	-	-	-	-	-	1	-	1	0.6
N	24	26	13	18	21	5	51	7	165	100%
N hap	2	1	1	5	5	3	8	6	15	

IA: Isla Aguada, CU: El Cuyo, CL: Las Coloradas, HX: Holbox, IC: Isla Contoy, AV: Aventuras DIF, XC: Xcacel-Xcacelito, SK: Sian Ka’an, N: Sample size, N hap: number of haplotypes, Hap freq: Haplotype frequency (%).

**Table 4:** Mitochondrial DNA control region haplotype frequencies (~817 bp fragment) for a green turtle foraging aggregation from Xcalak, Quintana Roo, Mexico (XQR).

Haplotype	XQR	Hap freq
CM-A1.1	5	19.2
CM-A2.1	1	3.8
CM-A3.1	18	69.2
CM-A5.1	1	3.8
CM-A13.1	1	3.8
N	26	100%
N hap	5	

N: sample size; N hap: number of haplotypes, Hap freq: Haplotype frequency (%).

The AMOVA results showed a maximum percentage of variation among groups (20%, *p* < 0.001; S7 Table) when grouping the nesting colonies from the Yucatán Peninsula into two MUs (scenario 2): 1) Mexican Caribbean, including all nesting colonies on the Quintana Roo coast, which we will now refer to as MCMX, and 2) Eastern Bay of Campeche, including nesting colonies from Campeche and Yucatán, which we will refer to as EBCMX as proposed before [49]. This result is also supported by a pairwise *F*_ST_ comparison, which shows high and significant values between the Mexican Caribbean and Campeche/Yucatán nesting colonies (S8 Table).

**Fig 3:**
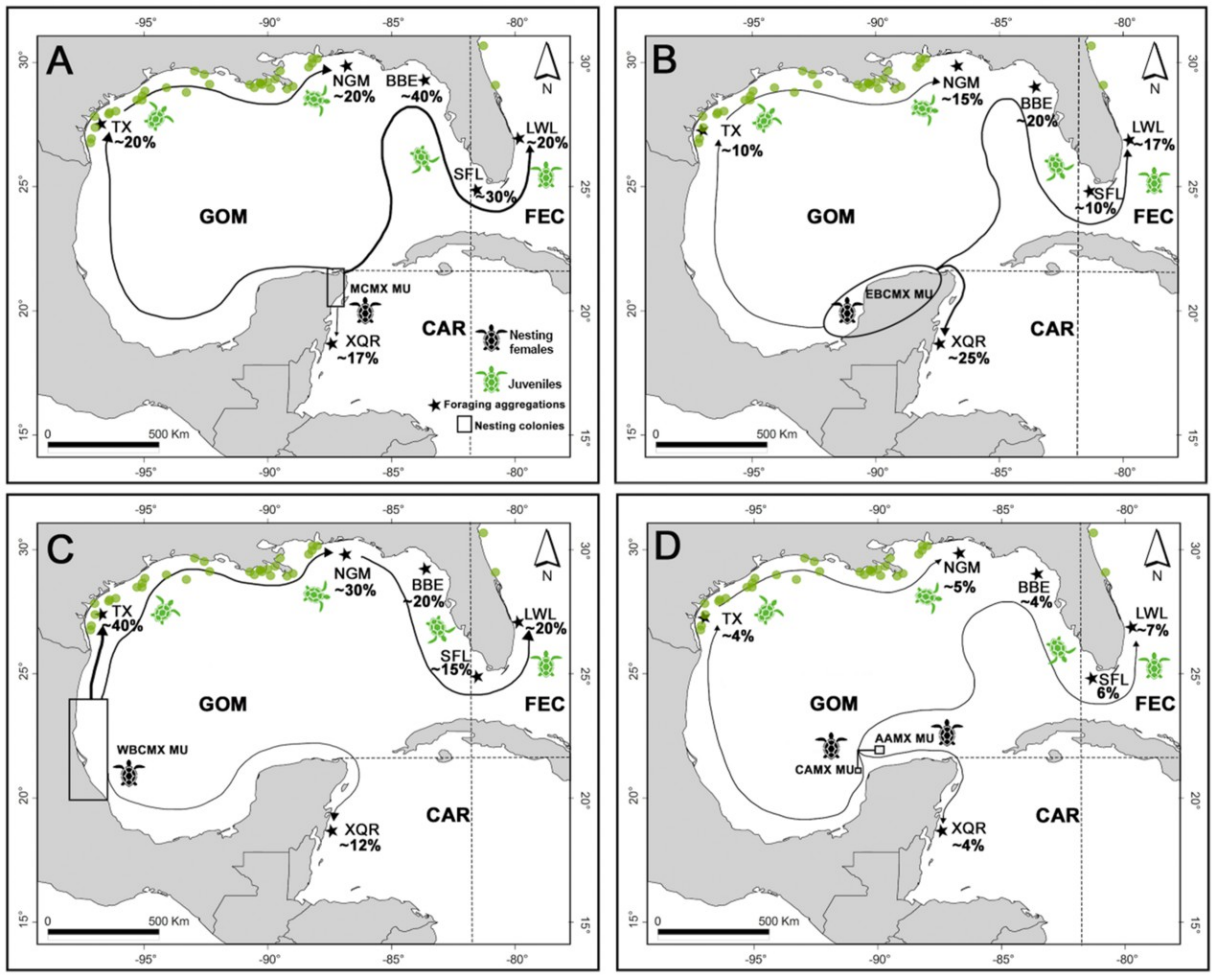
Migratory corridors in the Gulf of Mexico and Mexican Caribbean (black arrows) used by green turtles from Yucatán Peninsula nesting colonies, identified by MSA. Management Units are indicated by a geometrical shape (rectangle or oval): 3A) Mexican Caribbean MU (MCMX), 3B) Western Bay of Campeche MU (WBCMX), 3C) Eastern Bay of Campeche MU (EBCMX), and 3D) MUs from Cayo Arcas (CAMX) and Arrecife Alacranes (AAMX). Foraging aggregations are indicated by stars: Xcalak, Quintana Roo (XQR), Texas (TX), Northwestern Gulf of Mexico (NGM), Big Bend, Florida (BBE), South Florida, including Dry Tortugas and Everglades National Parks (SFL), and Lake Worth Lagoon, Florida (LWL). The contribution of each green turtle MU to foraging aggregations is indicated in percentage. The green dots indicate the geographic distribution of green turtle interactions with shrimp trawl fisheries in the NWA (available data at https://apps-sefsc.fisheries.noaa.gov/apex/f?p=112:20) to show the overlap between the fishing areas of the Gulf of Mex (GOM) and Florida east coast (FEC) and migratory corridors of green turtles in the NWA. The life stage of green turtles in nesting colonies and foraging aggregations is indicated by turtle color. For data on the size of turtles, see S9 Table. Based on data from direct observations compiled by the National Observer Program of the NOAA, there were 45 records of green turtles interacting with shrimp trawl fisheries between June 2009 and March 2021. The geographic distribution of these records (represented by green dots in Fig 3) revealed that the majority coincide with the migratory corridors identified in this study, particularly within the fishing areas of GOM and FEC (extending along the coasts of Texas, Louisiana, and Florida).

MSA based on the contribution of nesting colonies from the NWA grouping into seven MUs (S4 Table) satisfied the Gelman–Rubin criterion, reporting a value of 1.04. The MSA results showed that green turtles born in the MCMX MU predominantly traveled towards foraging aggregations in Florida (the contribution ranged from 20 to 40% between each locality), while a lesser proportion remained in the foraging aggregation in Xcalak (17%) displaying a local pattern of connectivity (Fig 3A). A high percentage of green turtles born in the WBCMX MU migrated to Texas (40%) and the Northwest of Gulf of Mexico (27%), and the remainder travelled to Florida and Xcalak (Fig 3B). Most individuals originating from EBCMX MU arrived in foraging aggregations in Xcalak (24%) and Florida (20%—distributed equally between each locality) (Fig 3C). Finally, the MUs from CAMX and AAMX contributed the lowest proportion of individuals (ranging from 4 to 7%) migrating to the foraging aggregations in the U.S. (Florida, Texas, and the Northwest of the Gulf of Mexico) and Xcalak (Fig 3D).

## Discussion

From a genetic perspective, our study offers insights that aid in identifying the migratory routes utilized by Mexican loggerheads and green turtles within the NWA. Additionally, we characterize, for the first time, the genetic composition of a foraging aggregation of juvenile green turtles in the Mexican Caribbean, contributing to a broader understanding of the spatial ecology of this species at both local and regional scales. In terms of conservation, our results suggest that bycatch from shrimp trawl fisheries could negatively impact Mexican loggerheads and green turtles, potentially leading to a decrease in future adult recruitment in their natal populations.

### Loggerhead turtles

Our results showed that loggerhead nesting colonies from Quintana Roo have a genetic composition dominated by CC-A2.1, which is the common haplotype found in both NWA and Mediterranean nesting colonies [43, 44, 50]. Other haplotypes previously reported in Mexico and Florida were found in lesser proportion (CC-A1.4, CC-A2.5, and CC-A10.1) [43, 44]. We did not find significant evidence of genetic differentiation between the nesting beaches in Quintana Roo. However, our results support previous findings that showed genetic structure between nesting colonies from Quintana Roo and those in the southeastern U.S. (Florida, South Carolina, and Georgia) [43, 51].

In this study, MSA results provide evidence on the connectivity patterns of loggerhead turtles from QRMX nesting colonies to the Northwest Atlantic, migrating along the corridors that extend from the Gulf of Mexico, the U.S. East Coast to Cape Hatteras in North Carolina. Some loggerhead turtles that hatched in the Mexican Caribbean can migrate even further north, ultimately reaching the North Atlantic subtropical gyre [52–55]. The migration pattern from south to north in loggerhead turtles within the NWA has been supported by several studies suggesting that this latitudinal movement is facilitated by oceanic currents [55, 56]. Regarding loggerhead turtles from QRMX, the Yucatán Current could facilitate the migration of individuals to development and foraging areas within the NWA. This assumption is plausible considering that the Yucatán Current crosses from the west of the Yucatán Channel and becomes the Loop Current once it reaches the Gulf of Mexico, serving as a direct migratory route from the Yucatán Channel to the Florida Straits [31, 53]. However, loggerhead turtles do not remain within the Gulf of Mexico but continue migrating toward the North Atlantic subtropical gyre [30, 52]. In this regard, our results support the evidence that the Gulf Stream System has a significant influence on sea turtle distribution and serves as a migratory corridor between southeast Florida and Cape Hatteras in North Carolina [56, 57]. Our results show that Mexican loggerhead turtles can migrate larger distances to fishing areas within the NWA, such as the NEC and NED.

Additionally, we report for the first time the presence of hybrid female loggerhead turtles in the Mexican Caribbean. Interspecific hybridization has been sporadically reported in sea turtle populations, primarily hybrids between loggerhead x hawksbill and loggerhead x green turtle [58]. The temporal and spatial overlap of different sea turtle species in the breeding areas can result in interspecific mating [59]. Studies in the northern Yucatán Peninsula have reported spatial overlap of the mating aggregations of loggerhead and green turtles [22]. It is also probable that hawksbill females coincide with loggerhead males in feeding areas in the Mexican Caribbean during the breeding and nesting season [22].

### Green turtles

Our results revealed that the haplotype composition of green turtle nesting colonies from the Yucatán Peninsula was dominated by the haplotype CM-A3.1, commonly detected in the nesting populations from Mexico and Florida [46]. We identified the haplotypes CM-A22.1 and CMA-29.1 in the Mexican Caribbean nesting colonies, clarifying the origin of these haplotypes previously reported as ’orphan’ in foraging aggregations from Texas and Big Bend, Florida, respectively [46, 48]. Similar to a previous study [60], we found a significant genetic differentiation between nesting colonies from the Mexican Caribbean and the Eastern Bay of Campeche (Campeche and Yucatán). Some factors such as the recent evolutionary history of sea turtle populations [51], the natal philopatry of females [61], and the oceanic current patterns could influence the genetic break between the Gulf of Mexico and the Mexican Caribbean [29, 62–64].

This study presents the first genetic analysis of a green turtle foraging aggregation in the Mexican Caribbean, located in Xcalak and primarily composed of juveniles (mean 56 cm CCL: curved carapace length). The predominant haplotype found was CM-A3.1, commonly reported in nesting populations across the western Atlantic [38, 65]. No endemic haplotypes from Mexican Caribbean nesting colonies were detected, supporting previous findings that suggest Mexican green turtles migrate towards the northeastern Gulf of Mexico and Florida for foraging [32, 47]. However, to verify this conclusion and expand our understanding of green turtle migratory routes from the Yucatán Peninsula, further genetic characterization of other foraging aggregations in the region is crucial.

The MSA results showed that migratory routes used by green turtles from the Yucatán Peninsula were determined by their natal nesting colony. Individuals from MCMX and EBCMX MUs travel to foraging aggregations in Florida, the northwestern Gulf of Mexico, and Texas, which agrees with other studies [32, 48, 55]. This migratory pattern is favored by the Yucatán Current as they follow the same migratory routes as Mexican loggerhead turtles [31, 66]. Studies based on satellite tracking and ocean models have reported that green turtles migrating from the Mexican Caribbean cross the Gulf of Mexico until they arrive and recruit at foraging aggregations located in Florida [30, 31, 36, 66]. On the other hand, green turtles from the WBCMX MU primarily travel towards the foraging aggregations in Texas and the northwestern Gulf of Mexico, outlining regional pattern connectivity from west to east [47, 55]. Dispersal simulation models of green turtle hatchlings suggest that individuals from the Gulf of Mexico recruit to the coasts of Texas and Louisiana, U.S., which supports our results [30]. This migration pattern is influenced by anticyclonic eddies, as well as surface currents that favor the retention of organisms within the Gulf of Mexico [67]. Previous research based on mitogenomic sequencing indicates that the nesting colonies originating from WBCMX are the primary contributors to the foraging aggregation in Texas [46]. Similar dispersal patterns of neritic juvenile Kemp’s ridley turtles (*Lepidochelys kempii*, Garman, 1880) corroborate this migratory corridor [30]. In addition, MSA revealed that green turtles from EBCMX and MCMX remained in the foraging aggregation to the south of Quintana Roo (XQR), near their natal beach, suggesting a local connectivity pattern. This pattern is consistent with the ’natal homing’ hypothesis, which proposes that juveniles tend to stay in feeding habitats close to their natal nesting colony [35].

### Potential bycatch impact on the sea turtle populations from the Yucatán Peninsula

The mapping of the spatial distribution of interactions between loggerhead turtles and shrimp trawl fisheries showed that the migratory corridors identified in the Gulf of Mexico and the U.S. East Coast have a high likelihood of interaction and risk of bycatch for loggerhead turtles. Historically, shrimp trawl fisheries have represented a significant threat to this species in the U.S. Southeast and Gulf of Mexico, as illustrated by the elevated number of interactions (23,300 per year) and deaths (647 per year) recorded in the NWA between 1990 and 2007 [68]. However, shrimp trawlers are not the only fishery that threatens loggerheads in this region; Finkbeiner et al. [68] concluded that at least 17 U.S. fisheries coincided spatially with critical habitats used by loggerheads within the NWA. While this study does not specifically identify whether other fisheries overlap with the migratory corridors used by Mexican loggerhead turtles, several studies have demonstrated that fisheries such as pelagic longlines, gillnets, and scallop dredges have also caused injuries and mortality in loggerhead turtles in this region [25, 27, 69, 70].

The impact of other fisheries, such as pelagic longlines, has been underrepresented in the NWA region due to insufficient data provided for this fishing gear [71]. However, from a global perspective, longline fisheries constitute the greatest anthropogenic source of mortality for loggerhead turtles in commercial fisheries in the Eastern Atlantic, Mediterranean Sea, and Western Africa [72–74]. Commercial longline fisheries have a high impact on sea turtle populations because of the high amount of fishing effort [75]. In the North Atlantic, effort data reported approximately 135 million hooks from the U.S. longline fleet from 1990 to 2009 [76]. In the Northeast Atlantic, Parra et al. [77] reported 18 different vessels resulting in 887,614 hooks during quarterly monitoring between 2015 and 2020 for Portuguese commercial longline fisheries.

The effects of bycatch on the population level can be quantified based on the magnitude of interactions, particularly mortalities, as well as the reproductive values of individuals caught [78]. In the U.S., trawling fisheries, including shrimp trawlers, cause the mortality of large juveniles and adults with higher reproductive values [70, 75]. Stewart et al. [27] found that large juveniles of loggerhead turtles (>63 SCL: straight carapace length) originating from Quintana Roo were caught in the northern fisheries areas in the NWA. This fact could lead to a decrease in adult recruitment to their natal breeding populations, thereby threatening the recovery and stability of the Mexican loggerhead turtle population.

On the other hand, for green turtles from the Yucatán Peninsula, when the spatial distribution of interaction data with shrimp trawl fisheries was mapped, we found that migratory corridors along the Gulf of Mexico, mainly along the coasts of Texas and Louisiana in the U.S., have potential bycatch risk within the NWA. Shrimp trawl fisheries in the Gulf of Mexico caused a high number of interactions (11,300 per year) and deaths (300 per year) for green turtles between 1990 and 2007 [68]. Particularly in Texas and Louisiana, brown shrimp trawl fisheries represent a threat of incidental catching for green turtles [79]. Likewise, green turtle bycatch and mortality have also been associated with other coastal fisheries, such as drift nets, longlines, and set nets [21].

Although reports regarding the size of green turtles caught in Gulf of Mexico fisheries are not available, it is likely that they are juveniles. This assumption is based on studies conducted in foraging aggregations in Florida and Texas, which reported a high abundance of oceanic juveniles (approximately 20–40 cm SCL) [80–82]. In addition, stranding records in the northern Gulf of Mexico during 2015–2019 showed that the green turtles stranded were mainly juveniles (size mean 37.9 ± 15.1 cm SCL) [83]. However, a clear correlation has not been established between these stranding records and shrimp trawl fisheries due to the lack of available information on spatial-temporal fishery effort [83]. A study on fisheries in the Mediterranean concluded that juvenile green turtles were more susceptible to bycatch in fishery operations because the fishing effort was higher in habitats used by juveniles, increasing the vulnerability to entanglement in drift nets [84]. This observation coincides with our assumption that younger green turtles are more easily caught in trawls along their migratory routes. Thus, shrimp trawl fisheries in the NWA have severe implications for the recovery and conservation of green turtle nesting colonies from the Yucatán Peninsula.

While it is true that large-scale fisheries were considered the primary cause of sea turtle bycatch, recent attention has been focused on small-scale fisheries, which can have an impact on bycatch levels [75, 85, 86]. In the Yucatán Peninsula, Cuevas et al. [13] recorded that more than 600 small-scale fishing boats operate to the north and west of this region. Fisheries along the north coast of the Yucatán Peninsula primarily use longlines and have reported sea turtle bycatch estimates ranging from 0.53 to 0.72 individuals per 1,000 hooks [13]. However, gillnets were more extensively used on the west coast with a bycatch rate reported at an average of 0.53 individuals per m² of net per season, suggesting that gillnets have a greater negative impact due to the number of dead sea turtles caught [13]. Therefore, on a local scale, loggerhead and green turtles born in Mexican nesting colonies, are at risk in critical habitats near their natal beaches, which could reduce the local recruitment and jeopardize the recovery and stability of the Mexican populations [13, 87].

## Conservation perspectives

The NWA has been identified as a region where bycatch poses a particularly high threat to sea turtles [88]. Wallace et al. [88] concluded that the regional conservation status of the RMUs of loggerheads and green turtles in the NWA is categorized as Low Risk-High Threats. This category highlights that, although current sea turtle populations are large and stable, the high degree of threats—including bycatch—creates uncertainty about their future. Therefore, recovery and conservation plans are necessary to prevent significant population-level impacts from becoming evident [88]. In this sense, despite local conservation efforts, the Mexican loggerhead and green turtle populations continue to face global threats such as bycatch, particularly as their migratory corridors extend over vast distances and often cross international borders.

Regarding sea turtle bycatch in Mexican waters, some concrete actions that could be taken to quantify the sea turtle bycatch rate and identify potential areas of interaction are: i) implementing or reinforcing monitoring programs with on-board observers in the shrimp trawl fisheries off the Yucatán Peninsula, following international guidelines on bycatch and sustainable fisheries; ii) obtaining regional bycatch data directly from personal interviews with fishermen; and iii) encouraging citizen science studies to collect preliminary data sea turtle distribution (through development of digital resources) to identify zones with potential threats. The proposed actions represent practical steps toward better quantifying bycatch rates and identifying high-risk areas for sea turtle species from the Yucatán Peninsula. By implementing these strategies, we can enhance conservation efforts and contribute to the long-term protection of sea turtle populations and their habitats.

## Supporting information

S1 File Definition of the dataset for the Analysis of Molecular Variance (AMOVA) to identify the Management units (MUs) of loggerhead and green turtles from the Yucatán Peninsula. (PDF)

S1 Table Fishing areas from NWA (for details see Cramer and Adams, 1999) where have been caught loggerhead turtles genetically characterized using the longer fragment of the mtDNA control region (~800 bp). Haplotype frequencies of these individuals were considered in the MSA as ‘mixed stocks’ of loggerhead turtles from NWA.
(PDF)

S2 Table Management units (MUs) of loggerhead turtles from the NWA were considered as ’source populations’ in the MSA. Estimated nesting female abundance (estimated female number/year) was calculated for each nesting colony according to Seminoff et al. (2015) and subsequently for each MU. Name’s abbreviation of nesting colonies are, **AV**: DIF Aventuras, **XC**: Xcacel-Xcacelito, **SK**: Sian Ka’an (include the nesting beaches: Cahpechen, Kanzul and Lirios Balandrin in Quintana Roo, Mexico), **QRM**: mainland Quintana Roo (include the nesting beaches: Paamul, Aventuras DIF, Chemuyil, Xcacel, XelHa, Punta Cadena, Tankah, Kanzul, Cahpechen, and Lirios Balandrin in Quintana Roo, Mexico; for details see Shamblin et al. 2012), **ICZ**: Cozumel Island, **CAP**: Cape Island, South Carolina, **OSS**: Ossabaw Island, Georgia, **CAN**: Canaveral National Seashore, Florida, **MEL**: Melbourne Beach, Florida, **JUN**: Juno Beach, Florida, **FTL**: Ft. Laurderdale, Florida, **SGI**: St. George Island, Florida, **CSB**: Cape San Blas, Florida, **DTR**: Dry Tortugas, Florida, **CSL**: Cay Sal Bank, Bahamas, **KEY**: Keewaydin Island, Florida, **CSK**: Casey Key, Florida. Include the nesting beaches: Cahpechen, Kanzul and Lirios Balandrin in Quintana Roo, Mexico.
(PDF)

S3 Table Foraging aggregations of green turtles within the NWA, which have been genetically characterized using the longer fragment of mtDNA control region (~817 bp). Haplotype frequencies for these foraging aggregations were considered in the MSA as ‘mixed stocks’ of green turtles from NWA.
(PDF)

S4 Table Management units (MUs) of green turtles from the NWA were considered as ’source populations’ in the MSA. Estimated nesting female abundance (estimated female number/year) was calculated for each nesting colony according to Seminoff et al. (2015) and subsequently for each MU. Name’s abbreviation of nesting colonies are for Mexico: **HX**: Holbox, **IC**: Isla Contoy, **AV**: DIF Aventuras, **XC**: Xcacel-Xcacelito, **SK**: Sian Ka´an (include the nesting beaches: Kanzul and Cahpechen, Quintana Roo, Mexico), **IA**: Isla Aguada, **CU**: El Cuyo, **CL**: Las Coloradas, **RN**: Rancho Nuevo, **VER**: Veracruz (include the nesting beaches: Farallón, Coyotes, and El Llano, Veracruz, Mexico; for details see Millán-Aguilar 2009), **CA**: Cayo Arcas, **AA**: Arrecife Alacranes, and for Florida, U.S.: **CAN**: Canaveral National Seashore, **MEL**: Melbourne Beach, **HUT**: Hutch **JUN**: Juno Beach, **TEQ**: Tequesta, **SNG**: Singer Island, **BCR**: Boca Raton, **BRW**: Hillsboro, Pompano, and Lauderdale beaches, **MAR**: Key West,; **DTR**: Dry Tortugas.
(PDF)

S5 Table Analysis of Molecular Variance (AMOVA) testing different scenarios to define the MUs of the nesting colonies of loggerhead turtles from the Mexican Caribbean. For tested scenarios definition see S1 Fig.
(PDF)

S6 Table Pairwise fixation index *F*_ST_ comparison for loggerhead turtle nesting colonies in the Mexican Caribbean. *F*_ST_ value (below the diagonal; negative values were considered as 0) and *p* value (above the diagonal). The name´s abbreviation for each locality is shown in Table 1.
(PDF)

S7 Table Analysis of Molecular Variance (AMOVA) testing different scenarios to define the MUs of the nesting colonies of green turtles from the Yucatán Peninsula. The highlighted scenario in bold showed the highest percentage of molecular variance. For tested scenarios definition see S2 Fig.
(PDF)

S8 Table Pairwise fixation index *F*_ST_ comparison for green turtle nesting colonies in the Yucatán Peninsula. *F*_ST_ values (below the diagonal; negative values were considered as 0) and *p* values (above the diagonal, significant values were shown in bold). The name´s abbreviation for each locality is shown in Table 1.
(PDF)

S9 Table Data on the size of individuals (female nesting or juveniles) from each site (management unit, fishing areas, and foraging aggregations) where we obtained genetic data for this study. CCL: curved carapace length; SCL: straight carapace length; SSCL: straight standard carapace length; SD: standard deviation; NA: data not available.
(PDF)

S1 Fig Proposed scenarios for delimitation of MUs of loggerhead nesting colonies from Quintana Roo, Mexico. **Scenario 1:** *Quintana Roo* MU (black dots) including the four sampled sites (AV, XC, SK, and ICZ), **Scenario 2**: consider two MUs, *Mainland Quintana Roo* MU (red dots) including AV, XC, and SK, and *Insular Quintana Roo* MU (black dot) with ICZ. Localities names: **AV** Aventuras DIF, **XC** Xcacel-Xcacelito, **SK** Sian Ka’an, and **ICZ** Cozumel Island.
(PDF)

S2 Fig Proposed scenarios for delimitation of MUs of green turtles from the Yucatán Peninsula, Mexico. **Scenario 1:** *Yucatán Peninsula* MU (black dots) considering only one MU. **Scenario 2**: consider two MUs, *Eastern Bay Campeche* MU (green dots) including IA, CL, and CU, and *Mexican Caribbean* MU (blue dots) including HX, IC, AV, XC, and SK. **Scenario 3**: consider three MUs, *Campeche* MU (red dot) including IA, *Yucatán* MU (green dots) including CL and CU, and *Quintana Roo* MU (blue dots) including HX, IC, AV, XC, and SK. **Scenario 4**: consider three MUs, *Gulf of Mexico* MU (red dot) including IA, *northern Yucatán Peninsula* MU (green dots) including CL, CU, HX, and IC, and *Southern Yucatán Peninsula* MU (blue dots) including AV, XC, and SK. Localities names: ***Campeche***: **IA** Isla Aguada, ***Yucatán***: **CL** Las Coloradas and **CU** El Cuyo, ***Quintana Roo***: **HX** Holbox, **IC** Isla Contoy, **AV** Aventuras DIF, **XC** Xcacel-Xcacelito, and **SK** Sian Ka’an.
(PDF)

S3 Fig Nine U.S. fishing areas in the NWA (modified of Cramer & Adams, 1999). Names’ abbreviations are **MAB**: Mid -Atlantic Bight, **NEC**: Northeast Coastal, **NED**: Northeast Distant, **SAB**: South Atlantic Bight, **SAR**: Sargasso, **NCA**: north-central Atlantic, **GOM**: Gulf of Mexico, **FEC**: Florida East Coast, **CAR**: Caribbean.
(PDF)
