# Supplementary material for: Unraveling migratory corridors of loggerhead and green turtles from the Yucatán Peninsula and its overlap with bycatch zones of the Northwest Atlantic
Source: PLoS One. 2024 Dec 6;19(12):e0313685. doi: 10.1371/journal.pone.0313685 (PMC11623791; doi:10.1371/journal.pone.0313685)
Supplement: S1 File — (PDF) [file pone.0313685.s001.pdf]

In addition to the dataset generated in this study (N = 93), previously published haplotype frequencies (Shamblin et al., 2012; 2014) for nesting colonies from Cozumel Island (N = 21) and mainland Quintana Roo (grouping the following localities: Paamul, Aventuras DIF, Chemuyil, Xcacel, XelHa, Punta Cadena, Tankah, Kanzul, Cahpechen, and Lirios Balandrin; N = 156) were included in the AMOVA. Considering this complete dataset (N = 270) and considering the previous result that indicated only one population in this region (Shamblin et al., 2014), we proposed and tested two scenarios for nesting colonies in the Mexican Caribbean (1) a single MU grouping all nesting colonies present along the coast of Quintana Roo, and (2) two MUs which correspond to mainland vs insular nesting colonies (S1 Fig).

For green turtles, the AMOVA was carried out using the dataset generated for eight nesting colonies analyzed in this study (N = 165). To define the potential MUs, we tested for four possible scenarios: (1) all nesting colonies from the Yucatán Peninsula grouped in a single MU, (2) genetic segregation between nesting colonies from Campeche/Yucatán and Quintana Roo, (3) identification of three MUs separating the nesting colonies from Campeche, Yucatán, and Quintana Roo, and (4) genetic differentiation between nesting colonies from the northern and southern of the Yucatán Peninsula, and Campeche nesting colony (S2 Fig).

Shamblin BM, Bolten AB, Bjorndal KA, Dutton PH, Nielsen JT, Abreu-Grobois FA, et al. Expanded mitochondrial control region sequences increase resolution of stock structure among North Atlantic loggerhead turtle rookeries. Mar Ecol Prog Ser. 2012;469: 145-160.

Shamblin BM, Bolten AB, Abreu-Grobois FA, Bjorndal KA, Cardona L, Carreras C, et al. Geographic patterns of genetic variation in a broadly distributed marine vertebrate: new insights into loggerhead turtle stock structure from expanded mitochondrial DNA sequences. PLoS ONE. 2014;9: e85956.
