# Supplementary material for: Unraveling migratory corridors of loggerhead and green turtles from the Yucatán Peninsula and its overlap with bycatch zones of the Northwest Atlantic
Source: PLoS One. 2024 Dec 6;19(12):e0313685. doi: 10.1371/journal.pone.0313685 (PMC11623791; doi:10.1371/journal.pone.0313685)
Supplement: S1 Table — Haplotype frequencies of these individuals were considered in the MSA as ‘mixed stocks’ of loggerhead turtles from NWA. (PDF) [file pone.0313685.s002.pdf]

Abbrev	Fishing areas	N	Haplotype frequencies references
CAR	Caribbean	10	Stewart et al., 2018
GOM	Gulf of Mexico	59	Stewart et al., 2018
FEC	Florida east coast	28	Stewart et al., 2018
SAB	South Atlantic bight	28	Stewart et al., 2018
MAB	Mid Atlantic bight	163	Stewart et al., 2018
NEC	Northeast coastal	106	Stewart et al., 2018
SAR	Sargasso Sea	22	Stewart et al., 2018
NCA	North-central Atlantic	7	Stewart et al., 2018
NED	Northeast-Distant Atlantic	427	LaCasella et al., 2013; Stewart et al., 2018

N: Sample size

References for S1 Table

LaCasella EL, Epperly SP, Jensen MP, Stokes L, Dutton PH. Genetic stock composition of loggerhead turtles *Caretta caretta* bycaught in the pelagic waters of the North Atlantic. Endanger Species Res. 2013;22: 73-84.

Stewart KR, LaCasella EL, Roden SE, Jensen MP, Stokes LW, Epperly S, et al. Nesting population origins of leatherback turtles caught as bycatch in the U.S. pelagic longline fishery. Ecosphere. 2016;7: e01272.
