# Supplementary material for: Unraveling migratory corridors of loggerhead and green turtles from the Yucatán Peninsula and its overlap with bycatch zones of the Northwest Atlantic
Source: PLoS One. 2024 Dec 6;19(12):e0313685. doi: 10.1371/journal.pone.0313685 (PMC11623791; doi:10.1371/journal.pone.0313685)
Supplement: S2 Table — Estimated nesting female abundance (estimated female number/year) was calculated for each nesting colony according to Seminoff et al. (2015) and subsequently for each MU. Name’s abbreviation of nesting colonies are, AV: DIF Aventuras, XC: Xcacel-Xcacelito, SK: Sian Ka’an (include the nesting beaches: Cahpechen, Kanzul and Lirios Balandrin in Quintana Roo, Mexico), QRM: mainland Quintana Roo (include the nesting beaches: Paamul, Aventuras DIF, Chemuyil, Xcacel, XelHa, Punta Cadena, Tankah, Kanzul, Cahpechen, and Lirios Balandrin in Quintana Roo, Mexico; for details see Shamblin et al. 2012), ICZ: Cozumel Island, CAP: Cape Island, South Carolina, OSS: Ossabaw Island, Georgia, CAN: Canaveral National Seashore, Florida, MEL: Melbourne Beach, Florida, JUN: Juno Beach, Florida, FTL: Ft. Laurderdale, Florida, SGI: St. George Island, Florida, CSB: Cape San Blas, Florida, DTR: Dry Tortugas, Florida, CSL: Cay Sal Bank, Bahamas, KEY: Keewaydin Island, Florida, CSK: Casey Key, Florida. Include the nesting beaches: Cahpechen, Kanzul and Lirios Balandrin in Quintana Roo, Mexico. (PDF) [file pone.0313685.s003.pdf]

Abbrev MU	Management Unit (MU), Country	Female abundance for MU	Nesting colonies grouped in each MU	Haplotype frequencies references	Nesting female abundance references
QRMX	Quintana Roo, MX	420	AV, XC, SK, QRM, and ICZ	This study<br>Shamblin et al., 2012	FFyCM, 2018,<br>Shamblin et al., 2012
NUSA	North Carolina and Georgia, U.S.	409	CAP and OSS	Shamblin et al., 2012, 2014	Shamblin et al., 2012, 2014
CEFL	Central Eastern Florida, U.S.	1286	CAN and MEL	Shamblin et al., 2012, 2014	Shamblin et al., 2012, 2014
SEFL	Southeastern Florida, U.S.	667	JUN and FTL	Shamblin et al., 2012, 2014	Shamblin et al., 2012, 2014
NWFL	Northwestern Florida, U.S.	31	SGI and CSB	Shamblin et al., 2012, 2014	Shamblin et al., 2012, 2014
DSRL	Cay Sal, BA and Dry Tortugas, U.S.	139	DRT and CSL	Shamblin et al., 2012, 2014	Shamblin et al., 2012, 2014
KEY	Keewaydin Island Florida, U.S.	11	KEY	Shamblin et al., 2012, 2014	Shamblin et al., 2012, 2014
CKS	Case Key Florida, U.S.	45	CSK	Shamblin et al., 2012, 2014	Shamblin et al., 2012, 2014

References for S2 Table

Flora, Fauna y Cultura de México, A.C (FFyCM). Personal communication. September 2018.

Shamblin BM, Bolten AB, Bjorndal KA, Dutton PH, Nielsen JT, Abreu-Grobois FA, et al. Expanded mitochondrial control region sequences increase resolution of stock structure among North Atlantic loggerhead turtle rookeries. Mar Ecol Prog Ser. 2012;469: 145-160.

Shamblin BM, Bolten AB, Abreu-Grobois FA, Bjorndal KA, Cardona L, Carreras C, et al. Geographic patterns of genetic variation in a broadly distributed marine vertebrate: new insights into loggerhead turtle stock structure from expanded mitochondrial DNA sequences. PLoS ONE. 2014;9: e85956.
