# Supplementary material for: Unraveling migratory corridors of loggerhead and green turtles from the Yucatán Peninsula and its overlap with bycatch zones of the Northwest Atlantic
Source: PLoS One. 2024 Dec 6;19(12):e0313685. doi: 10.1371/journal.pone.0313685 (PMC11623791; doi:10.1371/journal.pone.0313685)
Supplement: S3 Table — Haplotype frequencies for these foraging aggregations were considered in the MSA as ‘mixed stocks’ of green turtles from NWA. (PDF) [file pone.0313685.s004.pdf]

Abbrev	Foraging agrgregation, Country	N	Haplotype frequencies references
XQR	Xcalak, Quintana Roo, Mexico	26	This study
BBE	Big Bend, Florida, U.S.	149	Chabot et al., 2021
DTO	Dry Tortugas National Park, Florida. U.S.	116	Naro-Maciel et al., 2017
EVP	Everglades National Park, Florida, U.S.	22	Naro-Maciel et al., 2017
LWL	Lake Worth Lagoon, Florida, U.S.	81	Gorham et al., 2016
TEX	Texas, U.S.	167	Shamblin et al., 2017
NGM	Northwestern Gulf of Mexico, U.S.	121	Shamblin et al., 2018

N: Sample size

References for S3 Table

Chabot RM, Welsh RC, Mott CR, Guertin JR, Shamblin BM, Witherington BE. A sea turtle population assessment for Florida's Big Bend, Northeastern Gulf of Mexico. Gulf Caribb Res. 2021;32: 19-33.

Gorham JC, Bresette MJ, Guertin JR, Shamblin BM, Nairn CJ. Green turtles (*Chelonia mydas*) in an urban estuary system: Lake Worth Lagoon, Florida. Fla Sci. 2016;79: 14-27.

Naro-Maciel E, Hart KM, Cruciata R, Putman NF. DNA and dispersal models highlight constrained connectivity in a migratory marine megavertebrate. Ecography. 2017;40: 586-597.

Shamblin BM, Dutton PH, Shaver DJ, Bagley DA, Putman NF, Mansfield KL, et al. Mexican origins for the Texas green turtle foraging aggregation: a cautionary tale of incomplete baselines and poor marker resolution. J Exp Mar Biol Ecol. 2017;488: 111-120.

Shamblin BM, Witherington BE, Hirama S, Hardy RF, Nairn CJ. Mixed stock analyses indicate population-scale connectivity effects of active dispersal by surface-pelagic green turtles. Mar Ecol Prog Ser. 2018;601: 215-226.
