# Supplementary material for: Unraveling migratory corridors of loggerhead and green turtles from the Yucatán Peninsula and its overlap with bycatch zones of the Northwest Atlantic
Source: PLoS One. 2024 Dec 6;19(12):e0313685. doi: 10.1371/journal.pone.0313685 (PMC11623791; doi:10.1371/journal.pone.0313685)
Supplement: S4 Table — Estimated nesting female abundance (estimated female number/year) was calculated for each nesting colony according to Seminoff et al. (2015) and subsequently for each MU. Name’s abbreviation of nesting colonies are for Mexico: HX: Holbox, IC: Isla Contoy, AV: DIF Aventuras, XC: Xcacel-Xcacelito, SK: Sian Ka´an (include the nesting beaches: Kanzul and Cahpechen, Quintana Roo, Mexico), IA: Isla Aguada, CU: El Cuyo, CL: Las Coloradas, RN: Rancho Nuevo, VER: Veracruz (include the nesting beaches: Farallón, Coyotes, and El Llano, Veracruz, Mexico; for details see Millán-Aguilar 2009), CA: Cayo Arcas, AA: Arrecife Alacranes, and for Florida, U.S.: CAN: Canaveral National Seashore, MEL: Melbourne Beach, HUT: Hutch JUN: Juno Beach, TEQ: Tequesta, SNG: Singer Island, BCR: Boca Raton, BRW: Hillsboro, Pompano, and Lauderdale beaches, MAR: Key West,; DTR: Dry Tortugas. (PDF) [file pone.0313685.s005.pdf]

Abbrev MU	Management Unit (MU), Country	Female abundance for MU	Nesting colonies grouped in each MU	Haplotype frequencies references	Nesting female abundance references
MCMX	Mexican Caribbean, MX	8539	HX, IC, AV, XC, and SK	This study	FFyCM, 2018; PRONATURA, 2018, Antele-Sangabriel, 2017
EBCMX	Eastern Bay of Campeche, MX	4431	IA, CU, and CL	This study, Millán-Aguilar, 2009	Guzmán and García, 2015; 2016, Guzmán, 2017; Cuevas et al., 2010.
WBCMX	Western Bay of Campeche, MX	7610	RN and VE	Millán-Aguilar, 2009	Millán-Aguilar, 2009, Shamblin et al., 2018
CAMX	Cayo Arcas, Campeche, MX	250	CA	Millán-Aguilar, 2009	Millán-Aguilar, 2009, Shamblin et al., 2018
AAMX	Arrecife Alacranes, Yucatán, MX	828	AA	Millán-Aguilar, 2009	Millán-Aguilar, 2009, Shamblin et al., 2018
CEFL	Central eastern Florida, U.S.	1012	CAN, MEL, and HUT	Shamblin et al., 2015	Shamblin et al., 2015
SOFL	Southern Florida, U.S.	622	JUP, TEQ, SNG, BCR, BRW, MAR, and DRT	Shamblin et al., 2015	Shamblin et al., 2015

References for S4 Table

Antele-Sangabriel W. Protección, manejo y conservación de tortugas marinas en el Parque Nacional Isla Contoy. Quintana Roo (MX): Parque Nacional Isla Contoy. Comisión Nacional de Áreas Naturales Protegidas (CONANP); 2017, México.

Cuevas E, González-Garza B, Segovia, AC, Sosa JE. Tortugas marinas: poblaciones y hábitats críticos. In Durán R, Méndez M, editors. Biodiversidad y Desarrollo Humano en Yucatán. México. CICY, PPD-FMAM, CONABIO, SEDUMA; 2010. pp. 262-263.

Flora, Fauna y Cultura de México, A.C (FFyCM). Personal communication. September 2018.

Guzmán HV, García, PA. Informe Técnico 2014 del Programa de Conservación de Tortugas Marinas en Laguna de Términos, Campeche, México. Contiene información de: 1. CPCTM Xicalango-Victoria, 2. CPCTM Chacahito, 3. CPCTM Isla Aguada y 4. Reseña estatal regional. Technical Report. Campeche (MX): Área Protección de Flora y Fauna Laguna de Términos. Comisión Nacional de Áreas Naturales Protegidas (CONANP), México; 2015.

Guzmán HV, García, PA. Informe Técnico 2015 del Programa de Conservación de Tortugas Marinas en Laguna de Términos, Campeche, México. Contiene información de: 1. CPCTM Xicalango-Victoria, 2. CPCTM Chacahito, 3. CPCTM Isla Aguada y 4. Reseña estatal regional. Technical Report. Campeche (MX): Área Protección de Flora y Fauna Laguna de Términos. Comisión Nacional de Áreas Naturales Protegidas (CONANP), México; 2016.

Guzmán HV. Informe Técnico 2016 del Programa de Conservación de Tortugas Marinas en Laguna de Términos, Campeche, México. Contiene información de: 1. CPCTM Xicalango-Victoria, 2. CPCTM Chacahito, 3. CPCTM Isla Aguada y 4. Reseña estatal regional. Technical Report. Campeche (MX): Área Protección de Flora y Fauna Laguna de Términos. Comisión Nacional de Áreas Naturales Protegidas (CONANP), México; 2017.

Millán-Aguilar O. Estructura genética poblacional de la tortuga verde, Chelonia mydas, en el Golfo de México determinada por análisis de secuencias del ADN mitocondrial. MSc Thesis, Universidad Nacional Autónoma de México, Mazatlán, Sinaloa, Mexico. 2009.

PRONATURA Península de Yucatán. Programa para la Conservación de las Tortugas Marinas. Personal communication. September 2018.

Shamblin BM, Bagley DA, Ehrhart LM, Desjardin NA, Martin RE, Hart KM, et al. Genetic structure of Florida green turtle rookeries as indicated by mitochondrial DNA control region sequences. Conserv Genet. 2015;16: 673-685.

Shamblin BM, Witherington BE, Hirama S, Hardy RF, Nairn CJ. Mixed stock analyses indicate population-scale connectivity effects of active dispersal by surface-pelagic green turtles. Mar Ecol Prog Ser. 2018;601: 215-226.
