# Supplementary material for: Unraveling migratory corridors of loggerhead and green turtles from the Yucatán Peninsula and its overlap with bycatch zones of the Northwest Atlantic
Source: PLoS One. 2024 Dec 6;19(12):e0313685. doi: 10.1371/journal.pone.0313685 (PMC11623791; doi:10.1371/journal.pone.0313685)
Supplement: S5 Table — For tested scenarios definition see S1 Fig. (PDF) [file pone.0313685.s006.pdf]

**Source of variation**	**d.f**	**Variance components**	**% variation**	**F-statistic**	***p*-value**
Scenario 1. All nesting colonies from Quintana Roo coast					
Among populations	4	-0.006	0.00	F_{ST}= 0.00	0.52
Within populations	263	0.391	100.00		
Scenario 2. Mainland vs insular nesting colonies					
Among populations	1	0.00013	0.03	F_{CT}= 0.00	0.59
Among populations within groups	3	-0.0008	-0.22	F_{SC}= 0.00	0.53
Whitin populations	261	0.3903	100.18	F_{ST}= 0.00	0.53
