# Supplementary material for: Unraveling migratory corridors of loggerhead and green turtles from the Yucatán Peninsula and its overlap with bycatch zones of the Northwest Atlantic
Source: PLoS One. 2024 Dec 6;19(12):e0313685. doi: 10.1371/journal.pone.0313685 (PMC11623791; doi:10.1371/journal.pone.0313685)
Supplement: S6 Table — FST value (below the diagonal; negative values were considered as 0) and p value (above the diagonal). The name´s abbreviation for each locality is shown in Table 1. (PDF) [file pone.0313685.s007.pdf]

| | AV | XC | SK | QRM | ICZ |
|---|---|---|---|---|---|
| **AV** | - | 0.492 | 0.669 | 0.814 | 0.870 |
| **XC** | -0.005 | - | 0.394 | 0.415 | 0.447 |
| **SK** | -0.021 | -0.001 | - | 0.204 | 0.686 |
| **QRM** | -0.013 | 0.000 | 0.009 | - | 0.243 |
| **ICZ** | -0.031 | -0.004 | -0.024 | 0.006 | - |
