# Supplementary material for: Unraveling migratory corridors of loggerhead and green turtles from the Yucatán Peninsula and its overlap with bycatch zones of the Northwest Atlantic
Source: PLoS One. 2024 Dec 6;19(12):e0313685. doi: 10.1371/journal.pone.0313685 (PMC11623791; doi:10.1371/journal.pone.0313685)
Supplement: S7 Table — The highlighted scenario in bold showed the highest percentage of molecular variance. For tested scenarios definition see S2 Fig. (PDF) [file pone.0313685.s008.pdf]

**Source of variation**	**d.f**	**Variance components**	**% variation**	**F-statistic**	***p*-value**
Scenario 1. All nesting colonies from the Yucatán Peninsula					
Among populations	8	0.043	15.59	F_{ST}= 0.15	< 0.001
Within populations	156	0.235	84.41		
**Scenario 2. Nesting colonies grouping in Eastern Bay Campeche and Mexican Caribbean**					
Among populations	1	0.061	20.20	F_{CT}= 0.20	0.010
Among populations within groups	6	0.009	3.17	F_{SC}= 0.03	0.038
Whitin populations	157	0.235	76.83	F_{ST}= 0.23	< 0.001
Scenario 3. Nesting colonies from Campeche, Yucatán, and Quintana Roo					
Among populations	2	0.048	16.27	F_{CT}= 0.16	0.034
Among populations within groups	6	0.013	4.50	F_{SC}= 0.05	0.043
Whitin populations	157	0.235	79.24	F_{ST}= 0.20	< 0.001
Scenario 4. Nesting colonies from south Yucatán Peninsula, north Yucatán Peninsula and Campeche					
Among populations	2	0.037	12.96	F_{CT}= 0.12	0.05
Among populations within groups	6	0.017	5.88	F_{SC}= 0.06	< 0.001
Whitin populations	156	0.235	81.15	F_{ST}= 0.18	< 0.001
