# Supplementary material for: Unraveling migratory corridors of loggerhead and green turtles from the Yucatán Peninsula and its overlap with bycatch zones of the Northwest Atlantic
Source: PLoS One. 2024 Dec 6;19(12):e0313685. doi: 10.1371/journal.pone.0313685 (PMC11623791; doi:10.1371/journal.pone.0313685)
Supplement: S8 Table — FST values (below the diagonal; negative values were considered as 0) and p values (above the diagonal, significant values were shown in bold). The name´s abbreviation for each locality is shown in Table 1. (PDF) [file pone.0313685.s009.pdf]

	IA	CU	CL	HX	IC	AV	XC	SK
**IA**	-	0.225	0.529	**0.008**	**0.020**	**0.040**	**0.001**	**0.001**
**CU**	0.048	-	0.999	**0.005**	**0.001**	**0.022**	**0.001**	**0.001**
**CL**	0.006	0.000	-	**0.023**	**0.041**	**0.047**	**0.001**	**0.001**
**HX**	0.152	0.277	0.187	-	0.505	0.591	0.072	0.054
**IC**	0.100	0.203	0.130	-0.009	-	0.408	0.052	0.058
**AV**	0.212	0.529	0.353	-0.016	-0.020	-	0.506	0.267
**XC**	0.193	0.261	0.211	0.033	0.041	-0.019	-	0.216
**SK**	0.522	0.701	0.566	0.130	0.180	0.057	0.025	-
