# Supplementary material for: Unraveling migratory corridors of loggerhead and green turtles from the Yucatán Peninsula and its overlap with bycatch zones of the Northwest Atlantic
Source: PLoS One. 2024 Dec 6;19(12):e0313685. doi: 10.1371/journal.pone.0313685 (PMC11623791; doi:10.1371/journal.pone.0313685)
Supplement: S9 Table — CCL: curved carapace length; SCL: straight carapace length; SSCL: straight standard carapace length; SD: standard deviation; NA: data not available. (PDF) [file pone.0313685.s010.pdf]

Loggerhead turtles					
**Life stage**	**Locality**	**Locality abbreviation**	**Mean size ± SD**	**Range size**	**Reference**
Nesting females	Quintana Roo MU	QRMX MU	SCL= 99.6 ± 8.0 cm	SCL = 81 to 119 cm	Zurita, 2015
Juveniles	North Atlantic Northeast Distant	NED	SCL = 56.7 cm	SCL = 35 to 72 cm	LaCasella et al. 2013
Small juveniles	North of Cape Hatteras	MAB, NEC, and NED	SCL = 55.8 ± 5.8 cm	NA	Stewart et al., 2018
Large Juveniles			SCL = 70.6 ± 8.0 cm	NA	Stewart et al., 2018
Small juveniles	South of Cape Hatteras	SAB, GOM, FEC, SAR, NCA, and CAR	SCL = 58.3 ± 5.7 cm	NA	Stewart et al., 2018
Large Juveniles			SCL = 71.1 ± 5.5 cm	NA	Stewart et al., 2018
**Green turtles**					
**Life stage**	**Locality**	**Locality abbreviation**	**Mean size ± SD**	**Range size**	**Reference**
Nesting females	Mexican Caribbean UM	MCMX MU	CCL = 106.9 ± 5.2 cm	CCL = 89 to 125 cm	Zurita, 2015
Nesting females	Eastern Bay of Campeche MU	EBCMX MU	SCL = 106 ±5.8 cm	SCL = 91 to 118 cm	This study
Nesting females	Western Bay of Campeche MU	WBCMX MU	NA	NA	
Nesting females	Cayo Arcas, Campeche	CAMX MU	NA	NA	
Nesting females	Arrecife Alacranes, Yucatán	AAMX MU	NA	NA	
Juveniles	Xcalak, Quintana Roo, MX	XQR	CCL =56 ± 11.6 cm	CCL = 33 to 77 cm	This study
Juveniles	Big Bend, Florida, U.S.	BBE	SSCL = 38.9 ± 9.7 cm	SSCL = 23.9 to 81.3 cm	Chabot et al., 2021
Juveniles	Dry Tortugas National Park, Florida, U.S.	DTO	SCL= 54.8 cm	SCL = 22.7 to 112.7 cm	Naro-Maciel et al., 2016

**Life stage**	**Locality**	**Locality abbreviation**	**Mean size ± SD**	**Range size**	**Reference**
Juveniles	Everglades National Park, Florida, U.S.	EVP	SCL =37.5 cm	SCL= 23.8 to 67.5 cm	Naro-Maciel et al., 2016
Juveniles	Lake Worth Lagoon, Florida, U.S.	LWL	SSCL = 40.4 ± 9.7 cm	SSCL = 24.6 to 62.3 cm	Gorham et al., 2016
Juveniles	Texas, U.S.	TEX	SCL = 36.6 ± 12.5 cm	SCL = 14 to 81.3 cm	Shamblin et al., 2017
Juveniles	Northwestern Gulf of Mexico, U.S.	NGM	SCL = 19.3 ± 7.1 cm	SCL = 14.1 to 27.4 cm	Shamblin et al., 2018

References for S9 Table

Chabot RM, Welsh RC, Mott CR, Guertin JR, Shamblin BM, Witherington BE. A sea turtle population assessment for Florida's Big Bend, Northeastern Gulf of Mexico. Gulf Caribb Res. 2021;32: 19-33.

Gorham JC, Bresette MJ, Guertin JR, Shamblin BM, Nairn CJ. Green turtles (*Chelonia mydas*) in an urban estuary system: Lake Worth Lagoon, Florida. Fla Sci. 2016;79: 14-27.

LaCasella EL, Epperly SP, Jensen MP, Stokes L, Dutton PH. Genetic stock composition of loggerhead turtles *Caretta caretta* bycaught in the pelagic waters of the North Atlantic. Endanger Species Res. 2013;22: 73-84.

Naro-Maciel E, Hart KM, Cruciata R, Putman NF. DNA and dispersal models highlight constrained connectivity in a migratory marine megavertebrate. Ecography. 2017;40: 586-597.

Shamblin BM, Dutton PH, Shaver DJ, Bagley DA, Putman NF, Mansfield KL, et al. Mexican origins for the Texas green turtle foraging aggregation: a cautionary tale of incomplete baselines and poor marker resolution. J Exp Mar Biol Ecol. 2017;488: 111-120.

Shamblin BM, Witherington BE, Hirama S, Hardy RF, Nairn CJ. Mixed stock analyses indicate population-scale connectivity effects of active dispersal by surface-pelagic green turtles. Mar Ecol Prog Ser. 2018;601: 215-226.

Stewart KR, LaCasella EL, Jensen MP, Epperly SP, Haas HL, Stokes LW, et al. Using mixed stock analysis to assess source populations for at-sea bycaught juvenile and adult loggerhead turtles (*Caretta caretta*) in the north-west Atlantic. Fish Fish. 2018;20: 239-254.

Zurita JG. Biología y Conservación de las tortugas marinas en Quintana Roo. PhD. Thesis, Universidad Nacional Autónoma de México, Mexico City, Mexico. 2015.
