# Supplementary figures and images for: Unraveling migratory corridors of loggerhead and green turtles from the Yucatán Peninsula and its overlap with bycatch zones of the Northwest Atlantic
Source: PLoS One. 2024 Dec 6;19(12):e0313685. doi: 10.1371/journal.pone.0313685 (PMC11623791; doi:10.1371/journal.pone.0313685)

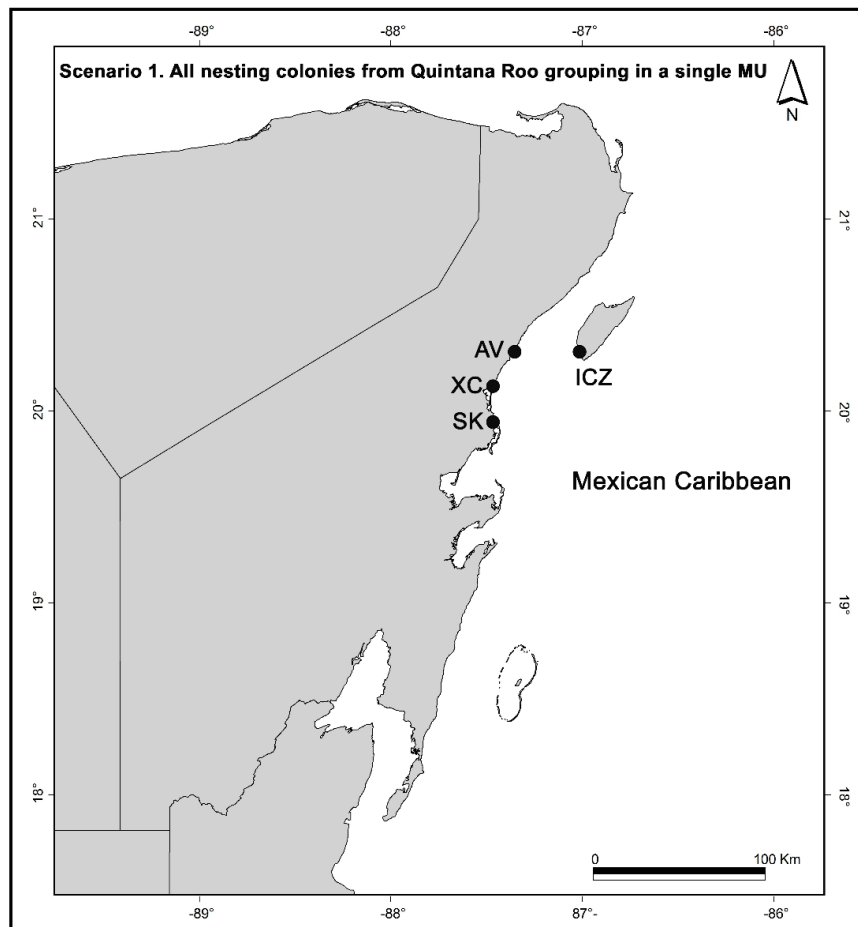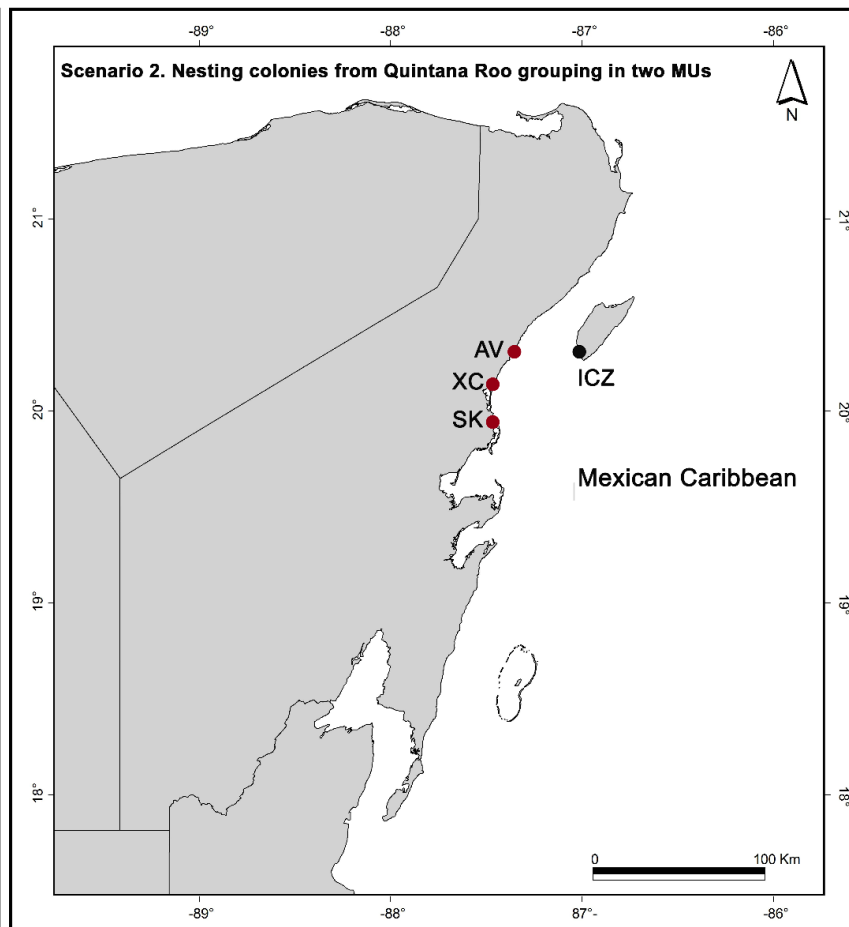

Supplement: S1 Fig — Scenario 1: Quintana Roo MU (black dots) including the four sampled sites (AV, XC, SK, and ICZ), Scenario 2: consider two MUs, Mainland Quintana Roo MU (red dots) including AV, XC, and SK, and Insular Quintana Roo MU (black dot) with ICZ. Localities names: AV Aventuras DIF, XC Xcacel-Xcacelito, SK Sian Ka’an, and ICZ Cozumel Island. (PDF) [file pone.0313685.s011.pdf]

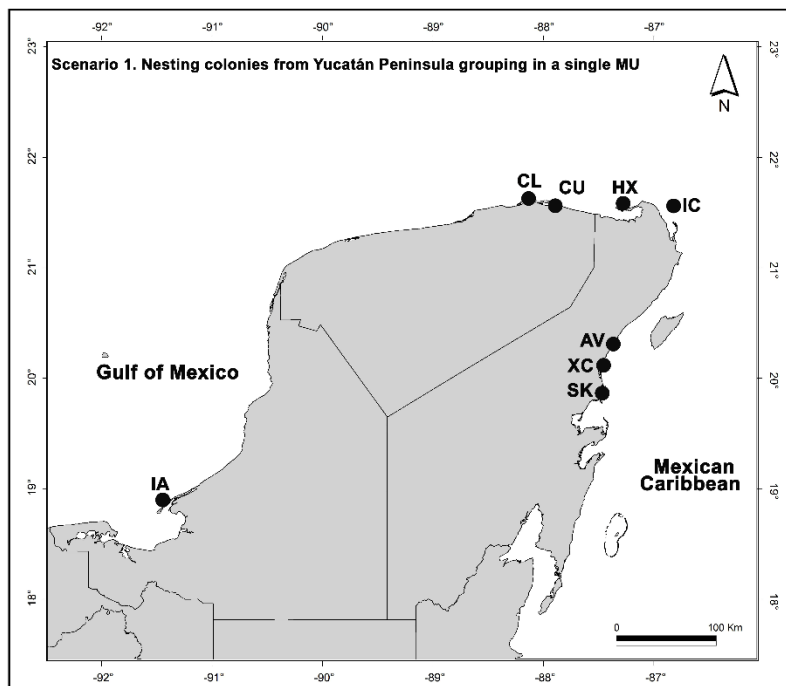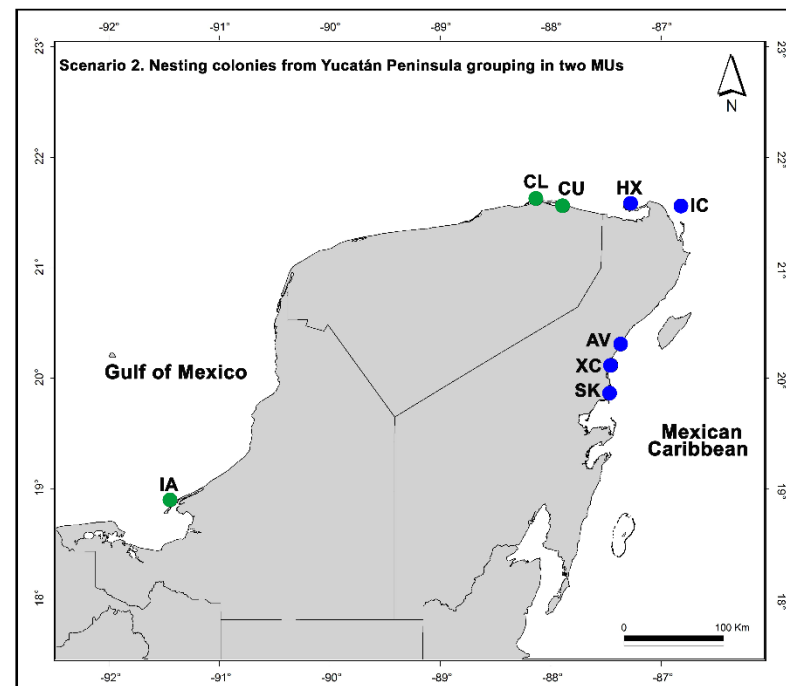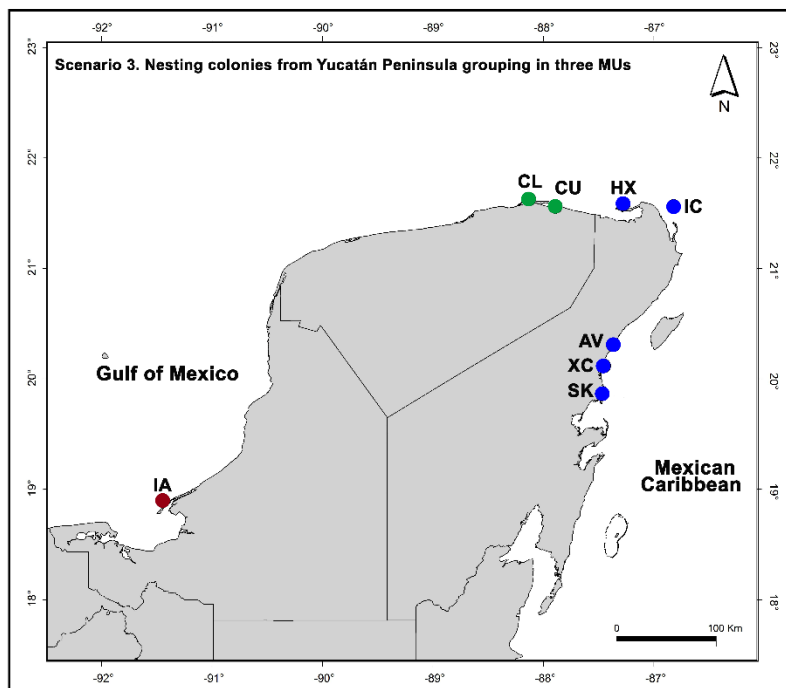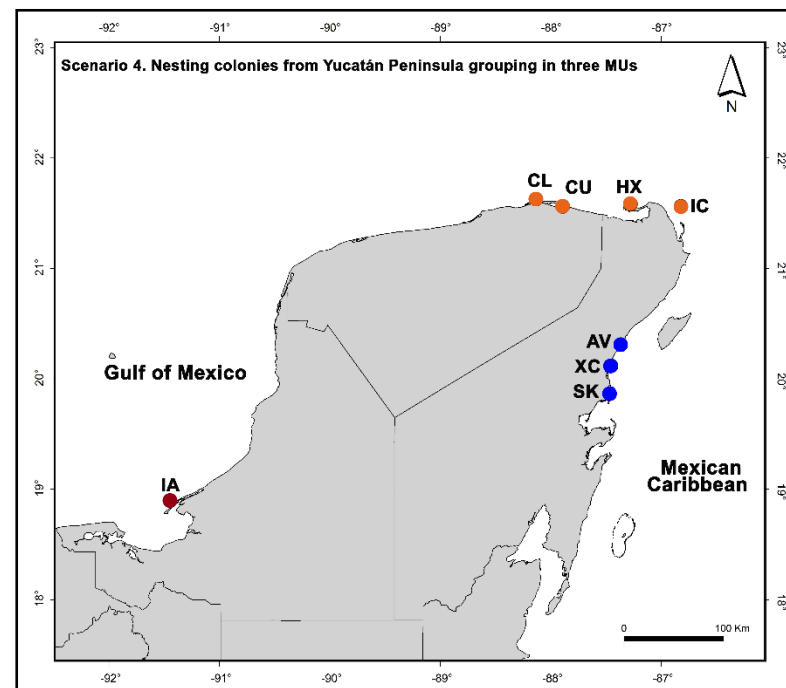

Supplement: S2 Fig — Scenario 1: Yucatán Peninsula MU (black dots) considering only one MU. Scenario 2: consider two MUs, Eastern Bay Campeche MU (green dots) including IA, CL, and CU, and Mexican Caribbean MU (blue dots) including HX, IC, AV, XC, and SK. Scenario 3: consider three MUs, Campeche MU (red dot) including IA, Yucatán MU (green dots) including CL and CU, and Quintana Roo MU (blue dots) including HX, IC, AV, XC, and SK. Scenario 4: consider three MUs, Gulf of Mexico MU (red dot) including IA, northern Yucatán Peninsula MU (green dots) including CL, CU, HX, and IC, and Southern Yucatán Peninsula MU (blue dots) including AV, XC, and SK. Localities names: Campeche: IA Isla Aguada, Yucatán: CL Las Coloradas and CU El Cuyo, Quintana Roo: HX Holbox, IC Isla Contoy, AV Aventuras DIF, XC Xcacel-Xcacelito, and SK Sian Ka’an. (PDF) [file pone.0313685.s012.pdf]

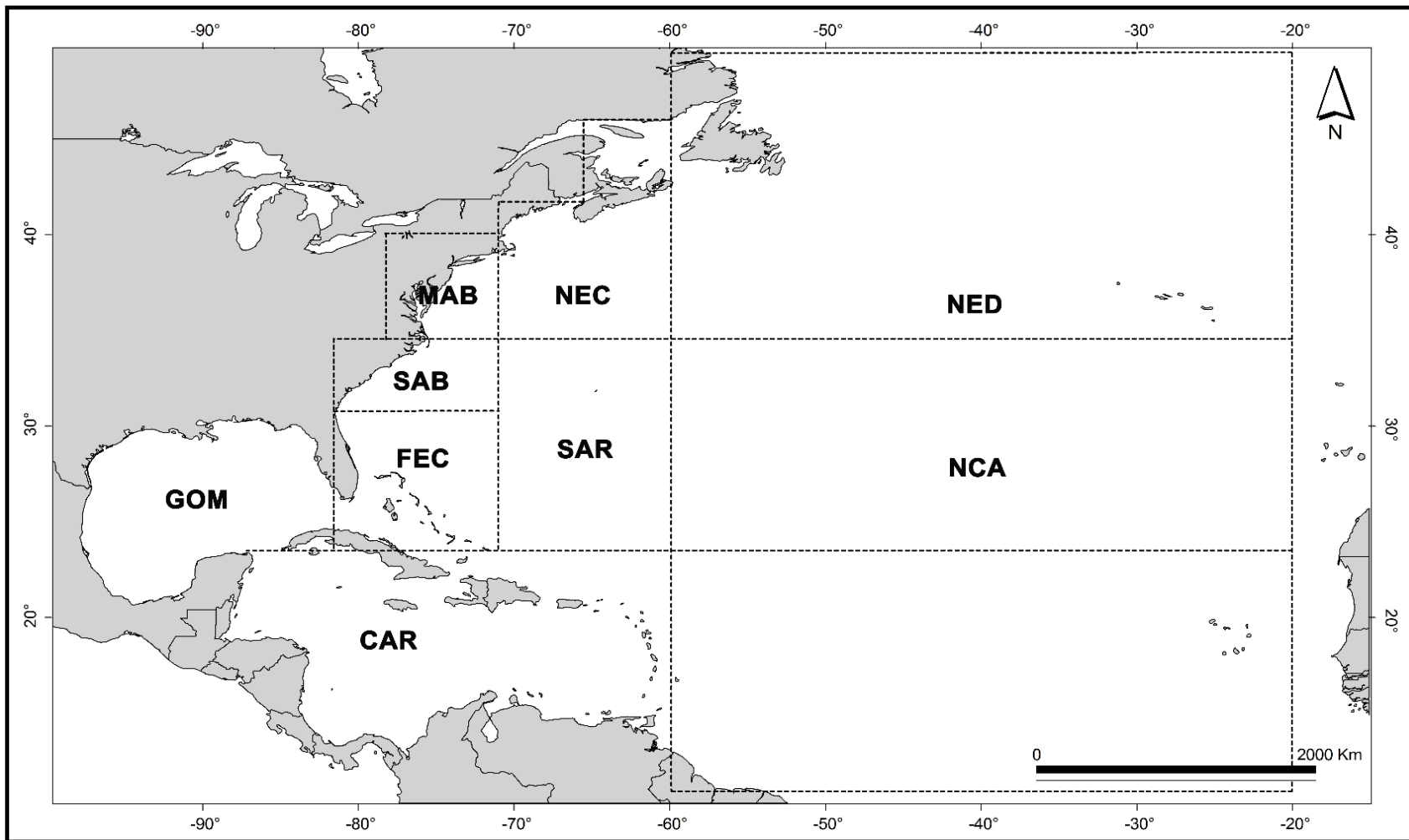

Supplement: S3 Fig — Names’ abbreviations are MAB: Mid -Atlantic Bight, NEC: Northeast Coastal, NED: Northeast Distant, SAB: South Atlantic Bight, SAR: Sargasso, NCA: north-central Atlantic, GOM: Gulf of Mexico, FEC: Florida East Coast, CAR: Caribbean. (PDF) [file pone.0313685.s013.pdf]
